# Construction and prognostic analysis of miRNA-mRNA regulatory network in liver metastasis from colorectal cancer

**DOI:** 10.1186/s12957-020-02107-z

**Published:** 2021-01-04

**Authors:** Ruyun Cai, Qian Lu, Da Wang

**Affiliations:** 1 Department of Colorectal Surgery, Sir Run Run Shaw Hospital, College of Medicine, Zhejiang University, Hangzhou, 310000 Zhejiang China; 2 Department of Medical Oncology, Sir Run Run Shaw Hospital, College of Medicine, Zhejiang University, Hangzhou, 310000 Zhejiang China

**Keywords:** Colorectal cancer, Liver metastasis, MicroRNA, Prognosis, Immune infiltration

## Abstract

**Background:**

Colorectal cancer (CRC) is one of the most common cancers in the world, and liver metastasis is the leading cause of colorectal cancer-related deaths. However, the mechanism of liver metastasis in CRC has not been clearly elucidated.

**Methods:**

Three datasets from the Gene Expression Omnibus (GEO) database were analyzed to obtain differentially expressed genes (DEGs), which were subjected to functional enrichment analysis and protein-protein interaction analysis. Subsequently, mRNA-miRNA network was constructed, and the associated DEGs and DEMs were performed for prognostic analysis. Finally, we did infiltration analysis of growth arrest specific 1 (GAS1)-associated immune cells.

**Results:**

We obtained 325 DEGs and 9 differentially expressed miRNAs (DEMs) between primary CRC and liver metastases. Enrichment analysis and protein-protein interactions (PPI) further revealed the involvement of DEGs in the formation of the inflammatory microenvironment and epithelial-mesenchymal transition (EMT) during the liver metastases process in CRC. Survival analysis demonstrated that low-expressed GAS1 as well as low-expressed hsa-miR-33b-5p was a favorable prognostic indicator of overall survival. Further exploration of GAS1 revealed that its expression was interrelated with the infiltration of immune cells in tumor tissues.

**Conclusions:**

In summary, DEGs, DEMs, and their interactions found in liver metastasis of CRC may provide a basis for further understanding of the mechanism of CRC metastasis.

## Introduction

Colorectal cancer is the third common cancer in the world with the second mortality rate [1]. It is estimated that there will be 147,950 new cases of CRC in the USA in 2020, and the CRC mortality rate from 2013 to 2017 was 13.9 per 100,000 people. Owing to the increasing availability of colonoscopy and improvements on treatment methods (such as surgical resection, chemotherapy, radiation therapy, and immunotherapy), there has been an increased number of early diagnoses and mitigation of mortality rates in CRC in singulos annos, whereas the rate of decline has slowed since the last 3 years [2, 3]. The primary cause of death in CRC is distant metastasis, which occurs in approximately 20% of patients at the time of initial diagnosis [4].

The liver is the most common site for distant metastases from CRC, which is probably due to the backflow of most mesenteric veins into the hepatic portal vein [5]. Liver metastasis occurs in approximately 25–30% of patients with CRC. Surgical resection is currently considered to be the most effective treatment for liver metastasis and can raise the 5-year survival rate to 50%, yet only about 25% of patients are suitable for surgical resection [6]. Understanding and in-depth research on the mechanism of metastasis is conducive to finding optimal therapies to liver metastasis in CRC. Although there is a lot of research on the molecular mechanisms of CRC liver metastasis, the definite mechanism and potential therapeutic targets are still unclear.

Single microRNA (miRNA) can target and regulate hundreds of mRNAs to participate in the regulation of cell proliferation, differentiation, and cell cycle [7]. miRNAs are single-stranded non-coding RNAs consisting of 21–23 nucleotides [8] and generally regarded as negative regulators of mRNA. Aberrant expression of some specific miRNAs in tumor cells has an impact on tumor progression and metastasis [9]. For example, miR-20a-5p promotes metastasis and invasion of CRC cells via targeting inhibition of drosophila mothers against decapentaplegic protein 4 (Smad4) expression [10]. miR-196a-5p plays a role in targeted modulation of phosphorylation of inhibitor α of NF-κB (IκBα) and its degradation, thereby making IκBα and nuclear factor-kappaB (NF-κB) unbind, eventually enhancing EMT of tumor cells [11, 12]. In CRC liver metastasis, miR-21 polarizes macrophages by targeting Toll-like receptor 7 to release more interleukin-6, which is involved in the composition of the liver inflammatory microenvironment suitable for colonization of metastatic tumor cells [13]. Prostate transmembrane protein androgen induced 1 (PMEPA1) has been shown to regulate the transforming growth factor-β signaling pathway to induce EMT in colorectal cancer cells, consequently promoting the occurrence of metastasis [14]. However, miR-378 may target and regulate PMEPA1 to reduce its expression, thus inhibiting the growth and invasion of CRC cells [15]. Many studies have also attempted to incorporate miRNAs into clinical diagnosis and therapeutic applications. For example, a set of biomarkers composed of 6 miRNAs (miR-21, let-7 g, miR-31, miR-92a, miR-181b and miR-203) can be used to diagnose CRC, with higher sensitivity and specificity than carcinoembryonic antigen (CEA) and carbohydrate antigen 19-9 (CA19-9) [16]. miR-655-3p and oxaliplatin can be directed into CRC liver metastases using nanoscale coordination polymers, thus inhibiting the growth of cancer cells. The abovementioned therapy regime implied that combining inhibitory miRNA with traditional chemotherapy can be used to stand a chance to treat metastatic tumors [17].

In this paper, we obtained eight miRNA-mRNA regulatory pairs by analyzing DEGs and DEMs between primary CRC and liver metastasis, which is of potential significance for further refining the evolutionary mechanism of liver metastasis. The miRNAs and mRNAs involved may develop into markers for auxiliary diagnosis. Further, it shows promise to be a targeted treatment option for metastatic CRC patients with poor efficacy of existing drug therapy in the coming period.

## Materials and methods

### Microarray dataset

The GEO [18] database was used to find gene expression data containing primary tumors and liver metastases of CRC. Two miRNA datasets and one genetic dataset were then selected and analyzed. The first miRNA dataset was GSE72199, based on the GPL15018 platform (Agilent-031181 Unrestricted_Human_miRNA_V16.0_Microarray 030840), containing 8 CRC liver metastases and 28 primary CRC lesions. The second miRNA data set, GSE54088, contained 9 CRC patients based on the GPL8178 platform (Illumina Human v1 MicroRNA expression beadchip). In consideration of the absence of some data and the fact that one of the patients suffered from both colon and rectal cancer, we eventually selected four paired primary CRC tumors and liver metastases data for analysis. The mRNA dataset was derived from GSE81582 based on the GPL15207 platform (Affymetrix Human Gene Expression Array), which contained 9 normal intestinal mucosae, 19 CRC liver metastases, and 23 primary CRC tissues. Finally, entire CRC liver metastases and primary CRC tissue data in GSE81582 dataset were included.

### Identification of DEMs and DEGs

GEO2R, an R-based online tool, was used for the analysis of DEMs and DEGs. The inclusion criteria for both DEGs and DEMs were *p* < 0.05 and |logFC| ⩾ 1. Then, the “VennDiagram” package in the R software was used to identify the common DEMs in GSE72199 and GSE54088.

### Enrichment analysis of DEGs

Upregulated and downregulated DEGs were uploaded to Database for Annotation, Visualization and Integrated Discovery (DAVID) [19] for Gene Ontology (GO) functional analysis, respectively. In addition, enrichment analysis of Kyoto Encyclopedia of Genes and Genomes pathway was carried out in KEGG Orthology-Based Annotation System (KOBAS) [20]. The inclusion criteria for both GO and KEGG was *p* < 0.05.

### Construction of PPI network and the most significant model

A web-based online tool, Search Tool for the Retrieval of Interacting Genes (STRING) [21], containing a database of 9,643,763 gene sequences from 2031 organisms, was used to build protein-protein interaction (PPI) networks to analyze the connections between proteins. The top ten genes under each of the 12 algorithms (Density of Maximum Neighborhood Component, Maximal Clique Centrality, Maximum Neighborhood Component, Clustering coefficient, Betweenness, BottleNeck, Closeness, Degree, EcCentricity, Edge Percolated Component, Radiality, Stress) were then calculated by the cytoHubba plugin in the Cytoscape software (version 3.6.0), and those genes present in at least 6 algorithms were identified as hub genes. The most significant model was then screened out using the MCODE plugin.

### Potential target genes for DEMs

The miRNet database is a web-based tool to analyze the interactions between mRNA, miRNA, and lncRNA, which integrates information from 11 databases including miRTarBase, TarBase, miRecords, and others [22]. Based on the fact that miRNAs negatively regulate target genes by combining with the 3′ untranslated region of mRNA, we utilized miRNet to predict the target genes of DEMs.

### Validation of target DEGs expression levels

Considering that metastatic tumors are affected by the liver background, we further validated the expression levels of DEGs in primary tumors. We used the Gene Expression Profiling Interactive Analysis (GEPIA) [23] to analyze the differential expression of DEGs between primary tumors and normal intestinal mucosa. GEPIA brings together 9736 tumors and 8587 normal samples from two major databases, The Cancer Genome Atlas (TCGA) and Genotype-Tissue Expression (GTEx). |log2FC| > 1 and *p* < 0.05 were deemed as the thresholds for significant differences.

### Prognostic analysis of DEGs and DEMs

To further understand the influence of the above genes on tumor prognosis, we divided each DEG and DEM into the high-expression group and the low-expression group with the median as the boundary. Then, the survival curves of 8 genes were constructed by GEPIA. Since GEPIA had no information about miRNA, OncoLnc [24], an online database integrating the survival information and mRNA, miRNA, and lncRNA expression information from the TCGA database, was selected to build the survival curves of DEMs. *p* values < 0.05 were considered significant.

### Infiltration analysis of GAS1-associated immune cells

**Fig. 1:**
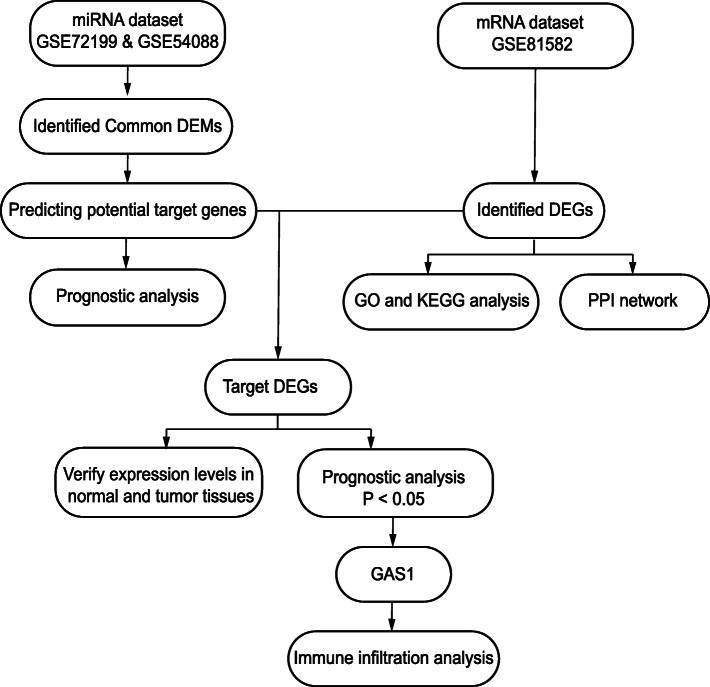
Flow chart of this paper

Tumor Immune Estimation Resource (TIMER) [25] is an online database that can be used to analyze immune infiltrates in different cancers. Therefore, we explored the correlation between GAS1 expression level and six different types of immune cells (including B cells, CD4+ T cells, CD8+ T cells, macrophages, myeloid dendritic cells (DCs), and neutrophils) in colon and rectal cancers, respectively. All the processes are shown in Fig. 1.

## Results

### Identification of DEMs and DEGs

**Fig. 2:**
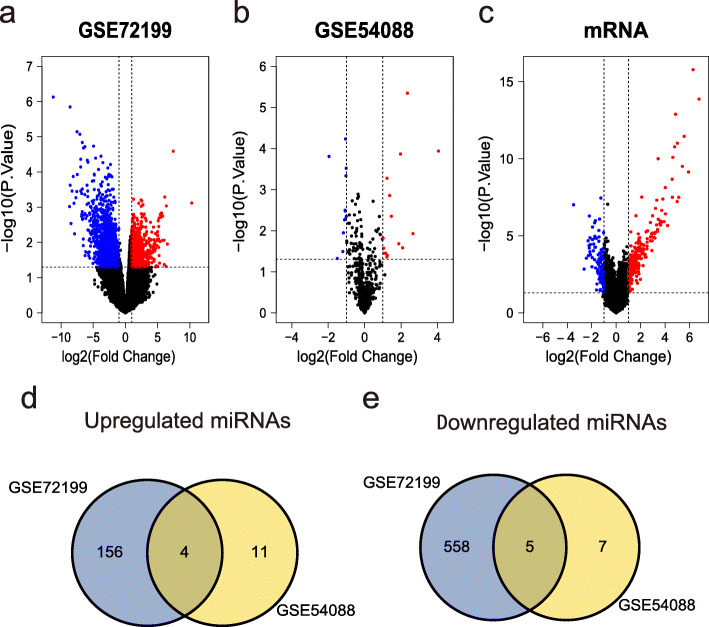
Volcano plots of **a** DEMs in GSE72199, **b** DEMs in GSE54088, and **c** DEGs in GSE81582 (GPL15207). The red color represents the upregulated genes between primary CRC tumors and liver metastases, while the blue color represents the downregulated genes, and the black indicates no significant difference. The Venn diagram shows the common **d** upregulated DEMs and **e** downregulated DEMs between GSE72199 and GSE54088. Blue represents the number of DEMs in GSE72199, yellow represents the number of DEMs in GSE54088, and the intersecting part represents the DEMs common to both data sets

To identify DEMs and DEGs between primary CRC and liver metastases, we analyzed two miRNA datasets (GSE72199 and GSE54088) and one mRNA dataset (GSE81582). According to the criteria of *p* < 0.05 and |logFC| ⩾ 1, we screened 842 DEMs including 160 upregulated DEMs and 563 downregulated DEMs in GSE72199, and 27 DEMs including 15 upregulated and 12 downregulated DEMs in GSE54088 (Fig. 2a, b). Subsequently, in accordance with above criteria, we identified 325 DEGs, including 219 upregulated DEGs and 106 downregulated DEGs (Fig. 2c).

In order to obtain the common upregulated and downregulated DEMs in the two miRNA datasets, we took their intersection through the Venn diagram. In the end, we got 4 upregulated DEMs and 5 downregulated DEMs (Fig. 2d, e, Table 1).

**Table 1:** Common differentially expressed miRNAs obtained from GSE72199 and GSE54088

miRNA	Expression change	GSE72199		GSE54088	
		LogFC	*P*-value	LogFC	*P*-value
hsa-miR-17-3p	Down-regulation	1.07	0.0274	1.06	0.0353
hsa-miR-33b-5p	Down-regulation	1.44	0.0425	1.23	0.0005
hsa-miR-210-3p	Down-regulation	1.22	0.0081	1.47	0.0045
hsa-miR-122-5p	Down-regulation	1.79	0.0254	4.05	0.0001
hsa-miR-576-5p	Up-regulation	-1.49	0.0229	-1.10	0.0054
hsa-miR-329-3p	Up-regulation	-1.26	0.0383	-1.06	0.0033
hsa-miR-152-3p	Up-regulation	-1.59	0.0315	-1.05	5.87E-05
hsa-miR-379-5p	Up-regulation	-1.11	0.0292	-1.03	0.0005
hsa-miR-137	Up-regulation	-3.52	0.0069	-1.01	0.0045

### GO functional analysis of DEGs

**Fig. 3:**
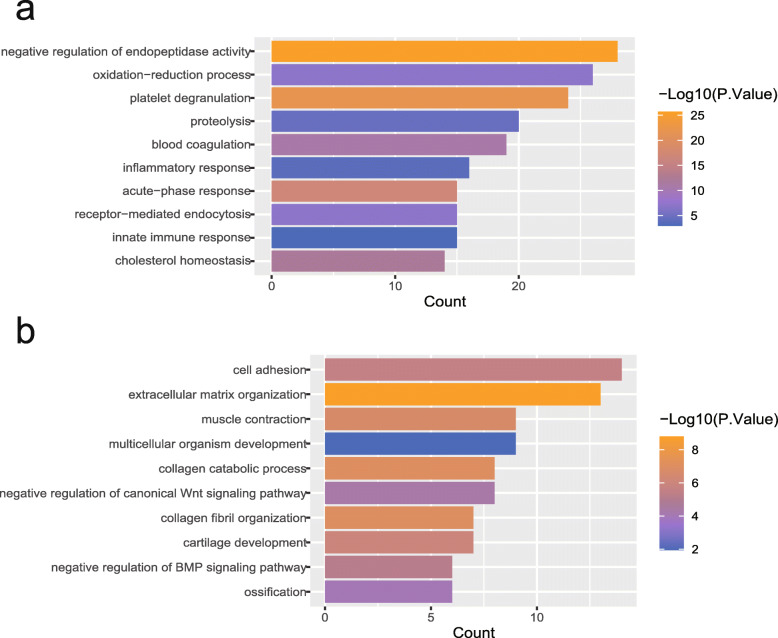
**a**The top 10 significant biological processes for upregulated DEMs. **b** The top 10 significant biological processes for downregulated DEMs. The color of the strip represents the *p* value; the closer the color is to orange, the smaller the *p* value is

For the sake of understanding the biological processes that DEGs participate in during CRC liver metastasis, we respectively uploaded the upregulated DEGs and downregulated DEGs to DAVID for GO functional analysis. The results show that upregulated DEGs are mainly involved in negative regulation of endopeptidase activity, platelet degranulation, blood coagulation, inflammatory response, acute-phase response, and innate immune response (Fig. 3a). As for those downregulated DEGs, they mainly modulate cell adhesion, extracellular matrix organization, muscle contraction, negative regulation of canonical Wnt signaling pathway, collagen fibril organization, and negative regulation of BMP signaling pathway (Fig. 3b).

### KEGG pathway enrichment analysis of DEGs

**Fig. 4:**
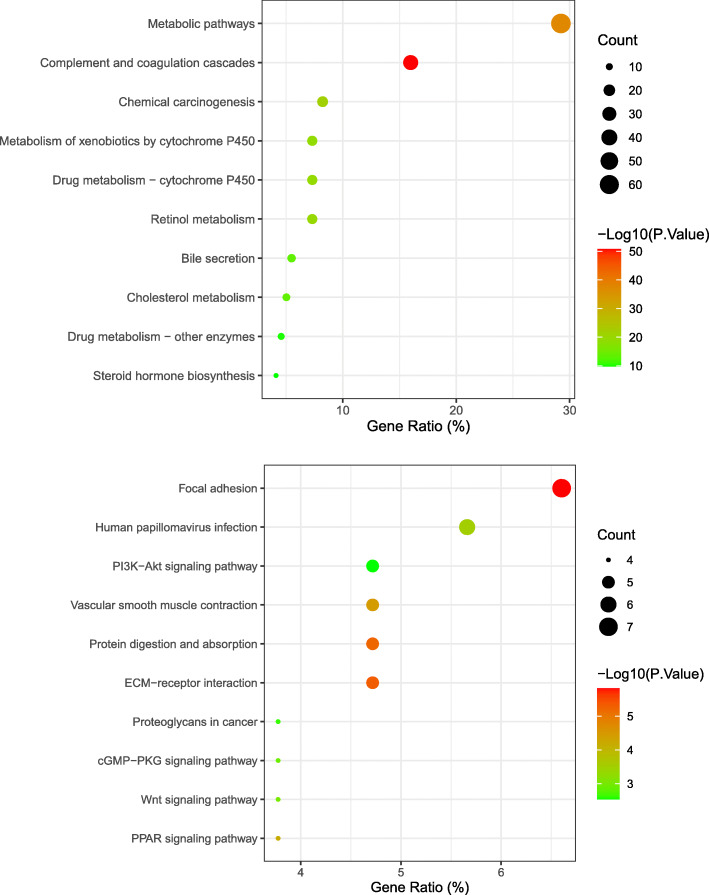
**a** The top 10 significant KEGG enrichment pathways in upregulated DEMs. **b** The top 10 significant KEGG enrichment pathways in downregulated DEMs. The node size represents the number of genes enriched in the pathway, and the *p* value of the red node is significantly lower than that of the green node

In the KEGG pathway enrichment analysis, we found that upregulated DEGs are mainly enriched in metabolic pathways, complement and coagulation cascades, chemical carcinogenesis, retinol metabolism, drug metabolism—cytochrome P450, metabolism of xenobiotics by cytochrome P450, bile secretion, cholesterol metabolism, drug metabolism—other enzymes, and steroid hormone biosynthesis (Fig. 4a). In terms of downregulated DEGs, the pathway enrichment analysis results focused on focal adhesion, human papillomavirus infection, ECM-receptor interaction, protein digestion and absorption, vascular smooth muscle contraction, PI3K-Akt signaling pathway, PPAR signaling pathway, Wnt signaling pathway, cGMP-PKG signaling pathway, and proteoglycans in cancer (Fig. 4b).

### Construction of PPI network and the most significant model

**Fig. 5:**
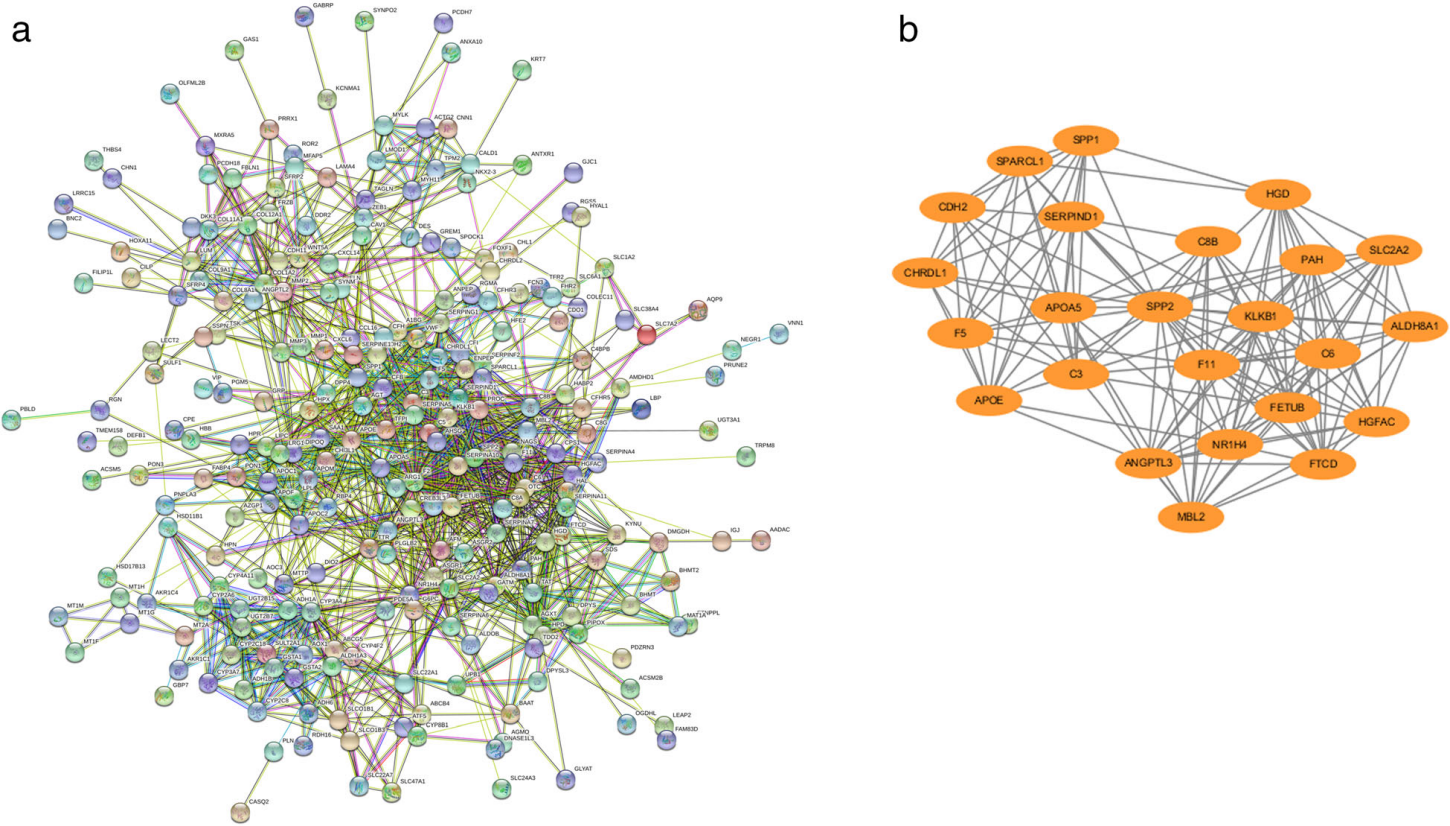
**a** Nodes in the PPI network represent proteins, while the edges between nodes represent interactions between proteins, and thicker lines indicate stronger connections. **b** The model with the highest score in PPI network, contains 24 nodes and 115 edges

We construct a PPI network containing 225 nodes and 1376 edges via STRING (Fig. 5a), with importing the results into the Cytoscape software on its heels. The first thing we did was to analyze the top ten genes under each of the 12 algorithms using the cytoHubba plugin, where nuclear receptor subfamily 1 group H member 4 (NR1H4), coagulation factor II, thrombin (F2), serpin family A member 10 (SERPINA10), and complement C3 (C3) were identified as hub genes that present in at least 6 algorithms. Then, the MCODE plugin was used, and the most remarkable model was obtained (Fig. 5b). For a deeper insight into the pathways involved in this model, we performed a KEGG pathway enrichment analysis, and the results are shown in Table 2.

**Table 2:** The enrichment pathway of the model for PPI network

Term	Count	*p* value
Complement and coagulation cascades	6	1.29E−12
Metabolic pathways	4	0.0030
Cholesterol metabolism	2	0.0002
*Staphylococcus aureus* infection	2	0.0004
Systemic lupus erythematosus	2	0.0015
Phagosome	2	0.002
Herpes simplex virus 1 infection	2	0.0186

### Potential target genes for DEMs

**Fig. 6:**
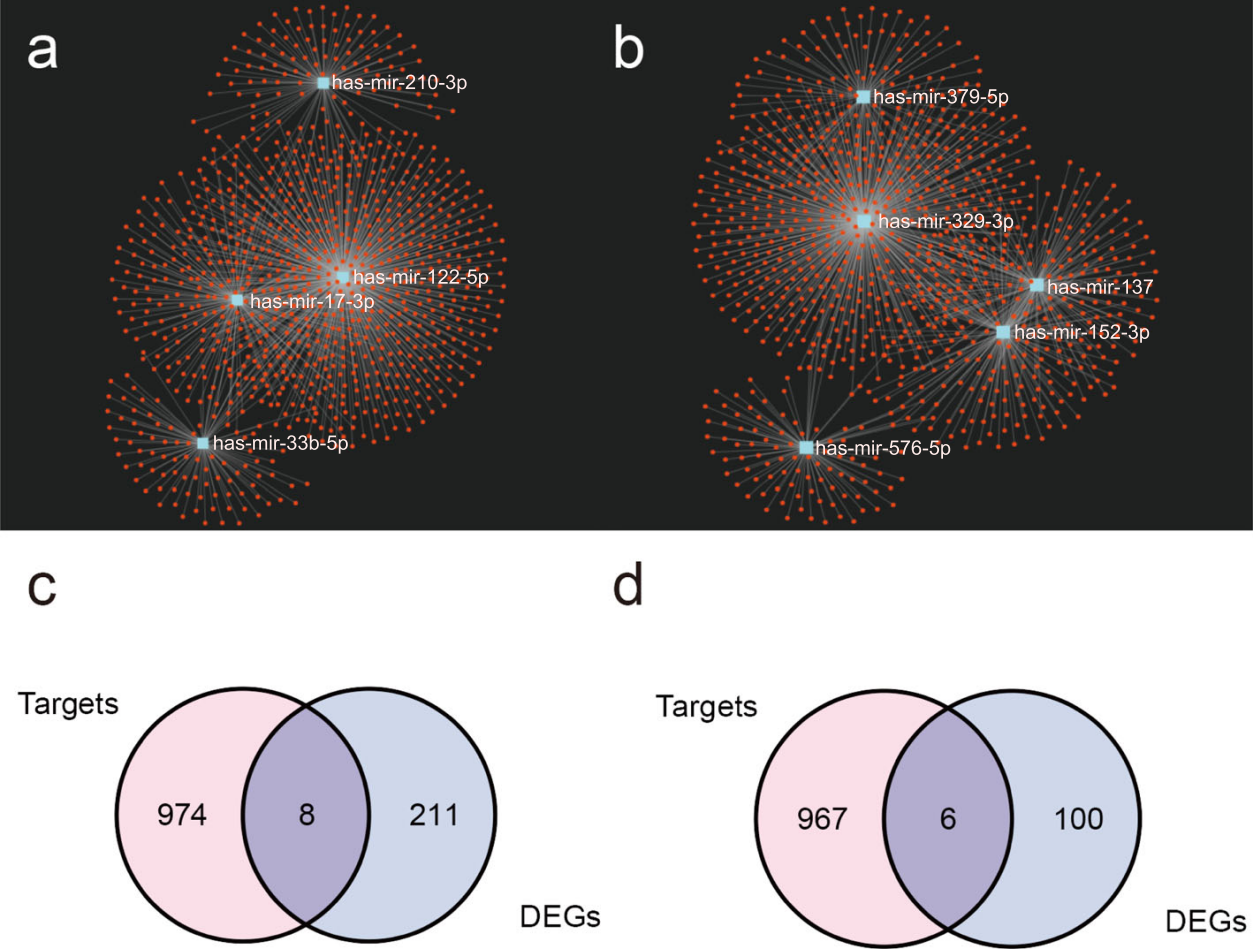
Potential target genes for **a** upregulated DEMs and **b** downregulated DEMs predicted using the miRNet database, respectively. The Venn diagram shows a common gene between the target genes and the DEGs. **c** The common genes between target genes of downregulated DEMs and upregulated DEMs. **d** The common genes between target genes of upregulated DEMs and downregulated DEMs. The pink color represents the number of target genes, the blue color represents the number of DEGs, and the purple in the middle represents the number of DEGs in common

For the purpose of predicting the target genes of DEMs, we used miRNet database to analyze the target genes of upregulated DEMs and downregulated DEMs, respectively. The network between DEMs and target genes was then demonstrated in miRNet’s built-in visualization (Fig. 6a, b). Immediately after, we used Venn diagrams to show common genes between target genes of downregulated DEMs and upregulated DEGs (Fig. 6c), and common genes between target genes of upregulated DEMs and downregulated DEGs were analyzed using the same method (Fig. 6d). Eight upregulated target genes (Table 3) and 6 downregulated target genes (Table 4) were obtained eventually.

**Table 3:** Target genes of upregulated miRNA

miRNA	Targets	Gene name
hsa-miR-17-3p	ENPP2	Ectonucleotide pyrophosphatase/phosphodiesterase 2
	BNC2	Basonuclin 2
hsa-miR-33b-5p	GAS1	Growth arrest-specific 1
	ZEB1	Zinc finger E-box binding homeobox 1
hsa-miR-122-5p	MYH11	Myosin heavy chain 11
	CALD1	Caldesmon 1

**Table 4:** Target genes of downregulated miRNA

miRNA	Targets	Gene name
hsa-miR-576-5p	CFHR3	Complement factor H-related 3
hsa-miR-329-3p	KYNU	Kynureninase
	SLCO1B3	Solute carrier organic anion transporter family member 1B3
	C6	Complement C6
	UGT2B4	UDP glucuronosyltransferase family 2 member B4
hsa-miR-379-5p	SERPINA1	Serpin family A member 1
hsa-miR-137	ASGR2	Asialoglycoprotein receptor 2
	SERPINA3	Serpin family A member 3

### Validation of target DEGS expression levels

**Fig. 7:**
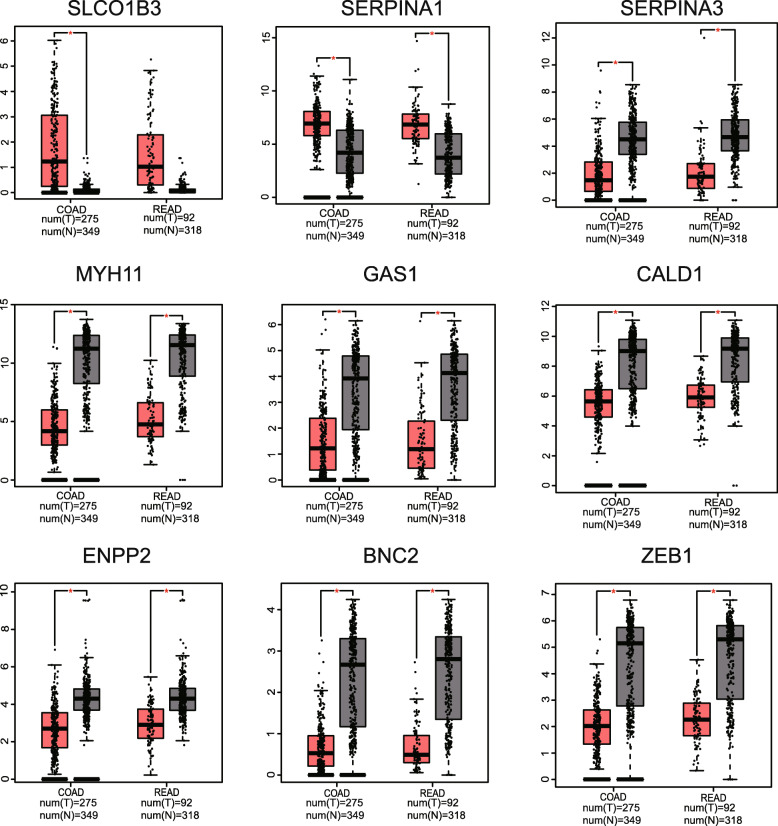
Expression level of DEGs between primary CRC tumors and normal intestinal mucosa in the GEPIA database. The graph on the left shows the differential expression of genes between colon cancer and normal colon mucosa. The right one shows the differential expression of genes between rectal cancer and normal rectal mucosa

The GEPIA database was thereafter utilized to validate the expression levels between 14 DEGs in CRC tissues and normal intestinal mucosa, and Fig. 7 shows 9 genes with significant expression differences. Of all the upregulated target DEGs, only the expression level of SERPINA3 in CRC was significantly lower than that in normal intestinal mucosa, which is contrary to what we observed in liver metastases. As for the downregulated target DEGs, it is visible that their expression levels in CRC are significantly reduced.

### Prognostic analysis of DEGs and DEMs

**Fig. 8:**
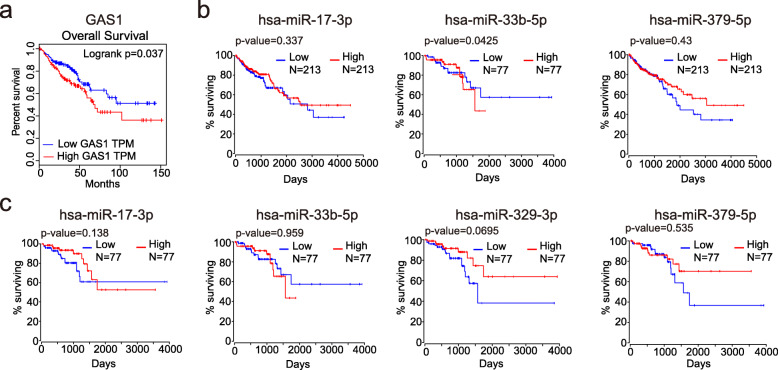
In all graphs, red represents the high expression group and blue represents the low expression group. **a** In the prognostic curves of GAS1 performed in GEPIA, the horizontal coordinate represents survival days, and the vertical coordinate represents overall survival rate, indicating that low expression group had better overall survival with *p* < 0.05. And the survival curves for DEMs in colon (**b**) and rectal (**c**) cancer were done separately using OncoLnc, with horizontal coordinate representing survival months and vertical coordinate representing overall survival rate. Results showed that only low expression of hsa-miR-33b-5p in colon cancer had a better prognosis with *p* = 0.0425

We divided differentially expressed genes into high and low expression groups based on their median expression level in colorectal cancer tumors and then analyzed the differences in survival time between the two groups. The results showed that only GAS1 had a significant difference in survival outcome between the high and low expression groups. Moreover, the high expression group had shorter overall survival and worse prognosis compared to the low expression group (HR = 1.6, Logrank *p* = 0.037) (Fig. 8a). Subsequently, we used the online database OncoLnc to analyze the effect of DEMs on survival time in colon cancer (Fig. 8b) and rectal cancer (Fig. 8c) separately. Similarly, DEMs were divided into high and low expression groups using the median expression level as a limit. Due to the low expression of hsa-miR-122-5p in colorectal cancer and the low expression of hsa-miR-329-3p in colon cancer, we were unable to obtain the survival data of these two miRNAs in corresponding tumors. The results showed that only the expression level of hsa-miR-33b-5p had a significant effect on the survival time of patients with colon cancer.

### Infiltration analysis of GAS1-associated immune cells

**Fig. 9:**
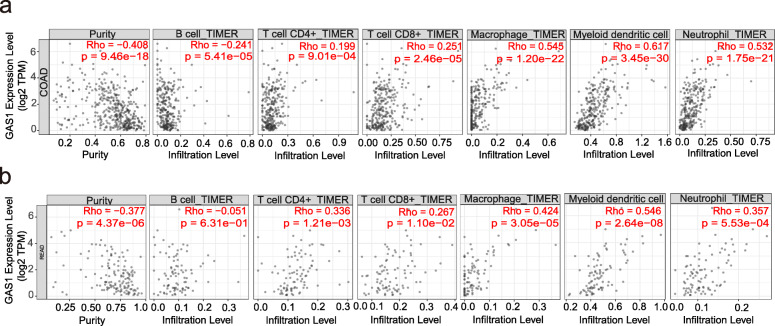
**a** Correlation between GAS1 expression level and the degree of immune cell infiltration in colon cancer tumors. **b** Correlation between GAS1 expression level and the degree of immune cell infiltration in rectal cancer tumors. *p* < 0.05 indicates a significant difference

Based on the results of DEGs survival analysis, we further investigated the relationship between GAS1 and immune cell infiltration in tumor tissues. Figure 9a and b respectively show the correlation between GAS1 and 6 immune cells in colon and rectal cancers after purity correction. The results showed that the low expression of GAS1 was negatively correlated with the distribution of CD4+ T cells, CD8+ T cells, macrophages, myeloid dendritic cells (DCs), and neutrophils in both colon and rectal cancer, but positively correlated with B cells.

## Discussion

CRC liver metastasis is influenced by multiple genes and pathways. Though lots of researches have been done in this field in recent years, the mechanism of its occurrence and development remains unclear. In this study, we firstly acknowledged 9 miRNAs (4 upregulated DEMs and 5 downregulated DEMs) and 325 mRNAs (219 upregulated DEGs and 106 downregulated DEGs) that were differentially expressed between primary CRC tumors and liver metastases. Next, analysis of GO-BP function and KEGG pathway enrichment revealed increased expression of genes involved in inflammatory responses, whereas expression of genes associated with cell adhesion and inhibition of WNT signaling pathways decreased. It has been reported that the persistence of chronic inflammation leads to genetic mutations that promote tumorigenesis; furthermore, the establishment of inflammatory microenvironment where immune cells produce some inflammatory factors also plays a vital role in the proliferation, invasion, and migration of tumor cells [26]. Further studies have found that inflammation and EMT not only affect and promote each other on the early stage of tumorigenesis, but also play a significant role in tumor metastasis. EMT can be regulated by a variety of signaling pathways to impair the adhesion between cells thus enhancing cell migration and metastasis [27].

Subsequently, the 4 hub genes, including NR1H4, F2, SERPINA10, and C3, of PPI network were analyzed. F2 as the precursor of thrombin plays a central role in the blood coagulation. Previous studies have uncovered that cancer is often accompanied by a blood hypercoagulable state and that patients with cancer tend to develop venous thrombosis compared with normal people [28]. During the evolution of CRC, venous thrombosis predicted a worse prognosis [29]. The complement system is one of the important components of the inflammatory response, and in recent years, the role of the complement system in cancer has gained much attention. C3 plays a crucial role both in the complement classical activation pathway and the bypass activation pathway. Studies have revealed that C3 is synthesized mainly by macrophages and the liver, meanwhile it has been found that crypt cells of normal intestinal mucosal epithelium, intestinal adenoma, and intestinal adenocarcinoma cells can also synthesize C3 more recently [30]. In a mouse model of ovarian cancer, C3 was found to promote tumor cell growth and tumor angiogenesis [31]. NR1H4 is a member of the nuclear receptor family, also known as the Farnesoid X receptor (FXR), which regulates the metabolism of bile acids, cholesterol, triglycerides, and glucose. Preceding studies have shown that FXR inhibits the proliferation and development of tumor cells in hepatocellular carcinoma [32]. And in colorectal cancer, upregulation of FXR can induce apoptosis [33]. In contrast, a recent study manifested that knocking out NR1H4 inhibited colon cancer cell proliferation and promoted apoptosis [34]. More experiments are needed to define the role of the NR1H4 in cancer. Serpin A10, encoded by SERPINA10 gene, is an anticoagulant. It has also been found to be highly expressed in pheochromocytoma, gastrointestinal neuroendocrine carcinoma, and corresponding liver metastases [35, 36].

Finally, through the prediction of target genes in DEMs from the miRNet database and validation of target gene expression levels by GEPIA, we obtained a miRNA-mRNA regulatory network consisting of hsa-miR-329-3p/SLCO1B3, hsa-miR-379-5p/SERPINA1, hsa-miR-122-5p/MYH11, hsa-miR-122-5p/CALD1, hsa-miR-33b-5p/GAS1, hsa-miR-33b-5p/ZEB1, hsa-miR-17-3p/ENPP2, and hsa-miR-17-3p/BNC2.

hsa-miR-329-3p is known to be a cancer suppressor gene, but is downregulated in a variety of tumor tissues. It has been reported that hsa-miR-329-3p can constrain tumor cell proliferation, migration, and invasion by upregulating associated apoptosis genes and downregulating associated anti-apoptosis genes [37, 38]. Related in vitro experiments by Li et al. demonstrated that restoration of hsa-miR-329-3p expression lifted the inhibitory effect on colorectal tumor cells. And low expression of hsa-miR-329-3p in CRC tissues and cell lines is associated with lymphatic metastasis [39], which suggests that hsa-miR-329-3p may be a biomarker for CRC metastasis. Regarding hsa-miR-379-5p, downregulation of its expression in gastric cancer is significantly associated with poor prognostic features such as lymph node metastasis and advanced TNM stage [40]. Whereas in prostate cancer, hsa-miR-379-5p expression upregulating was associated with EMT [41]. In in vitro experiments on CRC cells, high expression of hsa-miR-379-5p suppressed cell proliferation and migration [42].

Identifying with our results, studies have manifested that hsa-miR-122-5p expression level is higher in hepatic metastases than in primary CRC tumors. hsa-miR-122-5p may increase the risk of hepatic metastasis in colorectal cancer by regulating cationic amino acid transporter 1 (CAT1) expression [43]. Meanwhile, high levels of hsa-miR-122-5p in plasma may indicate colorectal cancer liver metastasis and be associated with shorter relapse-free survival (RFS) and overall survival (OS) time [44, 45]. Analysis of differential expression in the invasion front of CRC with early lymph node metastasis reveals high expression of hsa-miR-17-3p [46], which may enhance the proliferation and inhibit apoptosis of CRC cells by targeting prostate apoptosis response-4 (Par4) [47]. hsa-miR-33b-5p was shown to constrain tumor cell growth and induce cell cycle arrest, and decreased expression level in liver cancer and CRC, which is inconformity with the results of this paper [48, 49]. However, there is a study that shows high expression of miR-33b in lung cancer [50].

SLCO1B3 gene encodes organic anion transporting polypeptide 1B3 (OATP1B3) [51]. Several studies have found that SLCO1B3 is as well expressed in multifarious hormone-dependent cancers, such as prostate, testicular, and pancreatic cancer [52, 53]. SLCO1B3 overexpression in CRC can interfere with the P53 pathway, which may be the mechanism of chemotherapy resistance in P53 wild-type colorectal cancer [54]. Based on the finding of this paper, the negative regulation of hsa-miR-329-3p on SLCO1B3, it is expected to be a new target for the treatment for p53 wild-type patients with chemotherapy resistance. The protein encoded by SERPINA1 gene, is also known as human anti-trypsin (AAT), which mainly inhibits neutrophil elastase and other serine proteases. SERPINA1 was reported to be significantly elevated in the serum of CRC patients compared to healthy controls [55]. In addition, SERPINA1 modulates activity and invasiveness of tumor cells, which is associated with lymph node metastasis and poor prognosis [56]. The MYH11 gene encodes a contractile protein, myosin 11 [57]. Low expression of MYH11 has been reported to be associated with recurrence and metastasis of colorectal cancer [58]. Previous studies have shown that ZEB1 can be modulated by multiple pathways to promote tumor cell invasion and metastasis, playing a vital part in tumorigenesis and progression [59–61]. hsa-miR-33b-5p can attenuate EMT of tumor cells by targeting the modulation of ZEB1 in lung adenocarcinoma [62]. However, in the present study, the expression of ZEB1 was found to be significantly lower in liver metastases than in primary CRC tumors. This difference may be explained by the fact that primary tumors require greater metastatic capacity compared to liver metastases in which its metastatic capacity is receded because a new adaptive environment needs to be established. Caldesmon, encoded by Caldesmon 1 (CALD1) gene, is an actin regulatory protein distributed in smooth muscle and non-muscle cells, which can regulate smooth muscle contraction and cell movement [63, 64]. Existed research has indicated that CRC subtypes containing a large stromal component predicted higher relapse and metastasis rates [65]. CALD1 has been known to be specifically expressed in stromal cells, upregulated by transforming growth factor (TGF)-β signaling pathway, and was strongly associated with a shorter disease-free interval [66]. Accumulated studies on the CALD1 gene have shown that its alternative splicing was a pattern of colorectal cancer-specific alterations [67, 68]. CALD1 undergoes alternative splicing to produce two major protein subtypes, high (h-caldesmon) and low (l-caldesmon) molecular weight isoforms [69]. The reduction of l-caldesmon promotes the formation of podosome/invadopodia and the degradation of extracellular matrix, thus enhancing the invasiveness of cancer cells [70, 71]. Autotaxin (ATX) is encoded by ectonucleotide pyrophosphatase/phosphodiesterase family member 2 (ENPP2) gene, with the activity of lysophospholipase D, which can transform lysophosphatidylcholine (LPC) into lysophosphatidic acid (LPA) [72]. ATX/LPA signaling is involved in the development of many cancers and associated with tumor invasion and metastasis [73–75]. It has been discovered that ATX was rarely expressed in human colon cancer cell lines, such as HCT116, CACO-2, and SW480 [76]. However, in the early stage of colon cancer, high expression of ATX can promote tumor angiogenesis and increase the density of blood vessels [77]. Moreover, increased secretion of ATX by colon B cells will increase the risk of colitis and tumorigenesis [76]. But for all this, there was no systematic study to show the specific role of ATX during the genesis and development of CRC yet. In colorectal tumor cells, GAS1 can inhibit EMT by activating adenosine 5′-monophosphate (AMP)-activated protein kinase (AMPK) and inhibiting the mechanistic target of rapamycin kinase (mTOR) pathway; however, consistent with this paper, GAS1, as a tumor suppressor gene, is usually under-expressed in colorectal cancer, promoting metastasis of tumor cells and relating to recurrence of stages II and III colorectal cancer [78, 79]. The tumor microenvironment includes immune cells, tumor cells, and the surrounding stroma. The immune mediators produced by immune cells play a role in both tumorigenesis and progression [80]. In the infiltration analysis of immune cells in tumor tissues, it can be found that the expression level of GAS1 is related to the infiltration distribution of various immune cells. This suggests that GAS1 has the potential to be involved in the regulation of tumors through different types of immune cells. As for BNC2, we have not found researches on its role in the progression of CRC.

## Conclusions

In summary, we screened out the hub genes and constructed the miRNA-mRNA regulatory network by bioinformatics. Further studies showed that hsa-miR-33b-5p/GAS1 could affect the prognosis of patients with colorectal cancer, which may be beneficial to comprehend the molecular mechanisms of CRC liver metastasis and seek out new biomarkers for diagnosis and treatment.

## Data Availability

The datasets analyzed during the current study are available in the GEO database, https://www.ncbi.nlm.nih.gov/geo/query/acc.cgi?acc=GSE72199; https://www.ncbi.nlm.nih.gov/geo/query/acc.cgi?acc=GSE54088; https://www.ncbi.nlm.nih.gov/geo/query/acc.cgi?acc=GSE81582.

## Abbreviations

CRC: Colorectal cancer
GEO: Gene Expression Omnibus database
DEGs: Differentially expressed genes
DEMs: Differentially expressed miRNAs
EMT: Epithelial-mesenchymal transition
miRNAs: MicroRNAs
DAVID: Database for Annotation, Visualization and Integrated Discovery
KOBAS: KEGG Orthology-Based Annotation System
STRING: Search Tool for the Retrieval of Interacting Genes
PPI: Protein-protein interaction
GEPIA: Gene Expression Profiling Interactive Analysis
TCGA: The Cancer Genome Atlas
GTEx: Genotype-Tissue Expression
TIMER: Tumor Immune Estimation Resource
DCs: Myeloid dendritic cells
NF-κB: Nuclear factor-kappaB
IκBα: Inhibitor α of NF-κB
CEA: Carcinoembryonic antigen
CA19-9: Carbohydrate antigen 19-9
PMEPA1: Prostate transmembrane protein androgen induced 1
ATX: Autotaxin
